# Supplementary material for: Genomic loci mispositioning in Tmem120a knockout mice yields latent lipodystrophy
Source: Nat Commun. 2022 Jan 13;13:321. doi: 10.1038/s41467-021-27869-2 (PMC8758788; doi:10.1038/s41467-021-27869-2)
Supplement: Supplementary file 1 — Supplementary Information [file 41467_2021_27869_MOESM1_ESM.pdf]

Genomic loci mispositioning in *Tmem120a* knockout mice yields latent lipodystrophy

Rafal Czapiewski, Dzmitry G. Batrakou, Jose I. de las Heras, Roderick N. Carter, Aishwarya Sivakumar, Magdalena Sliwinska, Charles R. Dixon, Shaun Webb, Giovanna Lattanzi, Nicholas M. Morton, and Eric C. Schirmer

Supplementary Figures

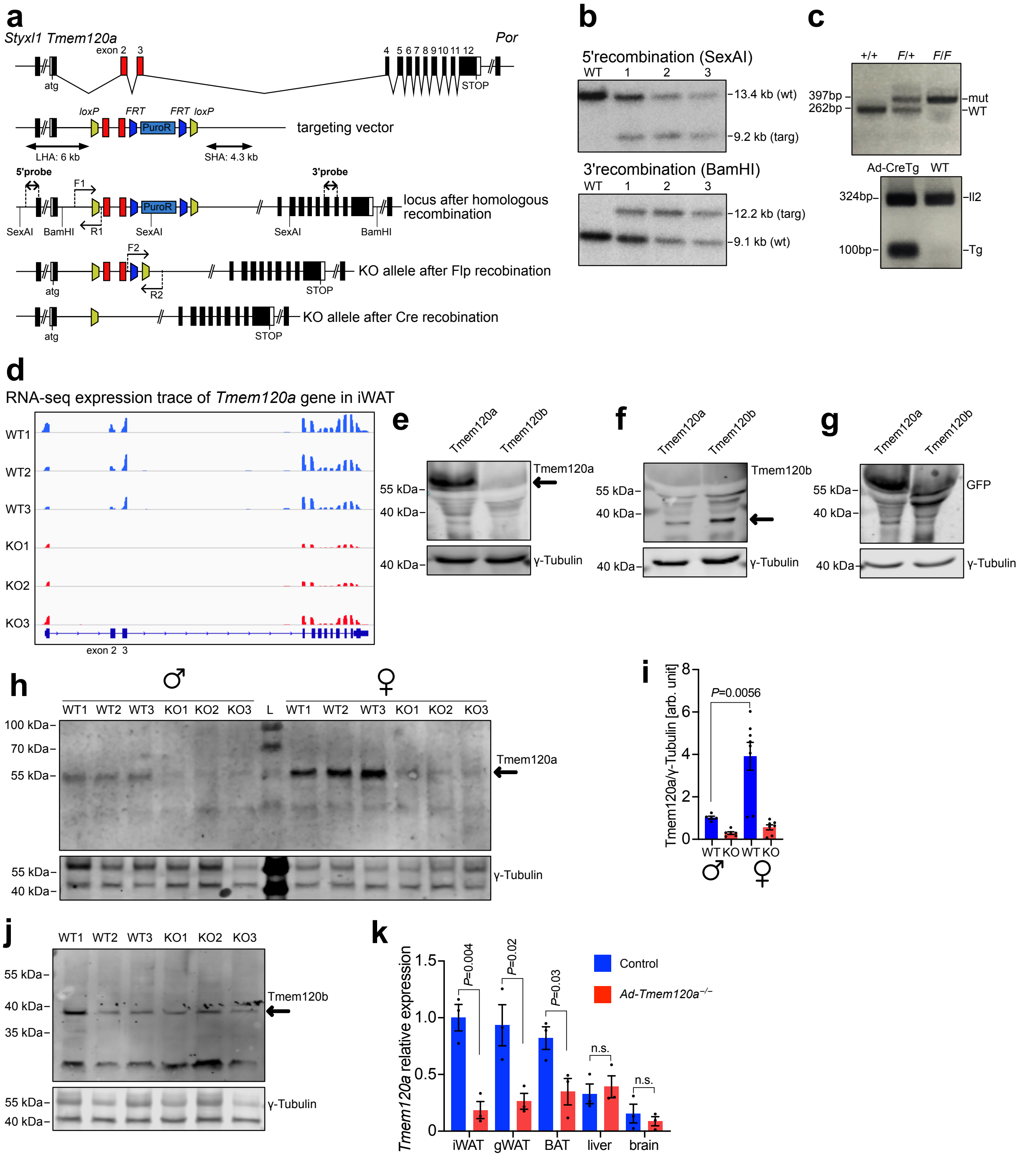

Supplementary Fig. 1

a-**c**, Strategy for adipose specific *Tmem120a*$^{-/-}$ mouse (*Ad-Tmem120a*$^{-/-}$). Exons 2 and 3 were flanked with *loxP* sites so upon expression of Cre recombinase under the adiponectin promoter *loxP* sites recombine leading to the loss of exon 2 and 3 - red boxes (**a**), the integration of targeting vector was confirmed by Southern blot with the use of 5' and 3' probes (**b**) and the animals were genotyped by PCR for the presence of the *loxP* sites and the presence of the Adiponectin *Cre* transgene – Tg (**c**). **d**, Representative RNA-Seq traces of Tmem120a gene expression on in iWAT showing lack of the exon 2 and 3 expression. **e-g**, Tmem120a (**e**) and Tmem120b (**f**) antibodies validation by Western blotting. HEK293T cells were transfected with plasmids carrying Tmem120 paralogs tagged with GFP. Both antibodies showed detection of expected size protein and expression of both proteins was further confirmed by GFP antibodies (**g**). **h and i**, Expression of Tmem120a protein in iWAT of male and female mice, controls vs. *Ad-Tmem120a*$^{-/-}$ shown by Western blotting (**h**) and quantification bar graph shown as mean and error bars show ±SEM from three experiments (n=3 per genotype). **j**, Representative Western blot showing the expression of Tmem120b paralog in iWAT from controls vs. *Ad-Tmem120a*$^{-/-}$. **k**, Relative expression of *Tmem120a* in subcutaneous inguinal fat by qPCR, n=3 animals per genotype/tissue measured in technical triplicates, shown as relative expression means and error bars show ±SEM. *P* values by unpaired two-sided Student's *t*-test. Source data are provided as a Source Data file.

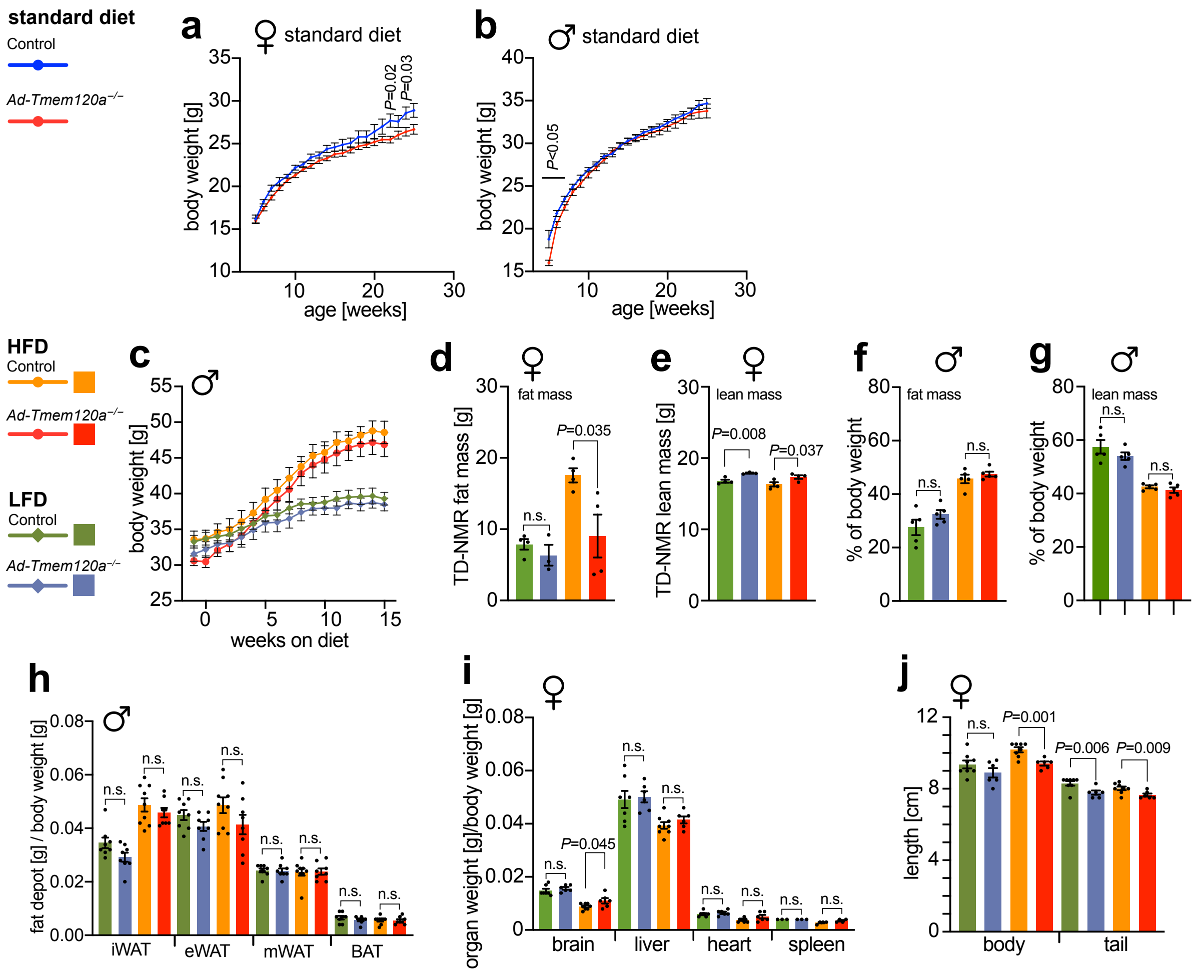

Supplementary Fig. 2

a and **b**, Body weight of mice on standard diet in females, control n=20, *Ad-Tmem120a*$^{-/-}$ n=29 (**a**), and in males, control n=29, *Ad-Tmem120a*$^{-/-}$ n=23 (**b**). **c**, Males body weight on high-fat (HFD) vs low-fat (LFD) diet, shown as mean and error bars show ±SEM (for each group n=10 per genotype, per diet). **d** and **e**, Non-normalised female mice body composition measured by TD-NMR after 10 weeks of LFD and HFD, fat mass (**d**) and lean mass (**e**) are shown as mean mass in grams; SEM (n=4). **f** and **g**, Male mice body composition measured by TD-NMR after 10 weeks of LFD and HFD, fat mass (**f**) and lean mass (**g**) are shown as percent of whole-body weight; shown as mean SEM (n=5 per genotype and diet). **h**, Whole body mass-normalised weight of different adipose tissue depots in males: inguinal-subcutaneous (iWAT), epididymal (eWAT), mesenteric (mWAT) white adipose tissue and inter-scapular brown adipose tissue (BAT), shown as mean ±SEM, control LFD n=8, *Ad-Tmem120a*$^{-/-}$ LFD n=8, control HFD n=9, *Ad-Tmem120a*$^{-/-}$ n=8. **i,** Profile of brain, liver, heart and spleen weight, normalised to body weight in female control and knockout animals on HFD and LFD (n=6-8) shown as mean and error bars show ±SEM. **j**, Length of body (nose to tail base) of animals on HFD is shorter statistically in KO, tail is shorter in both LFD and HFD in KO, shown as mean and error bars show ±SEM, control LFD n=8, *Ad-Tmem120a*$^{-/-}$ LFD n=6, control HFD n=8, *Ad-Tmem120a*$^{-/-}$ n=6. *P* values calculated by unpaired, two-sided Student's *t*-test. Source data are provided as a Source Data file.

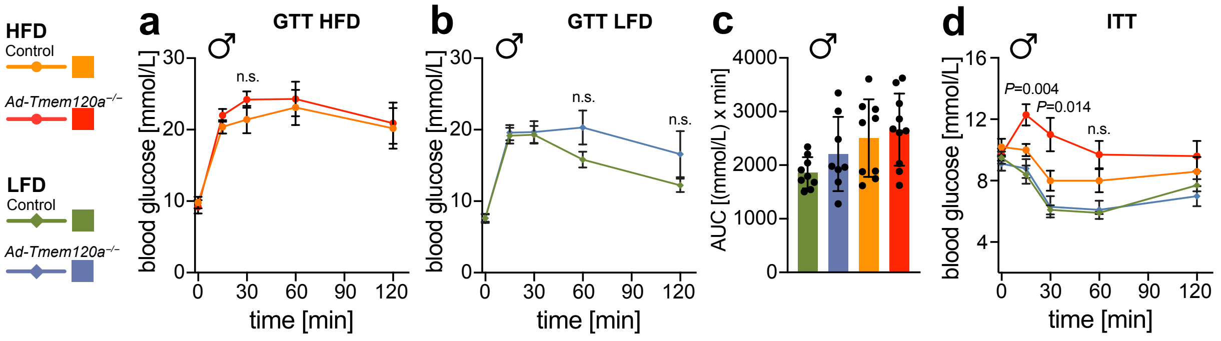

Supplementary Fig. 3

a-**c**, Glucose tolerance test (**GTT**) in control and *Ad-Tmem120a*$^{-/-}$ males on HFD (**a**) and LFD (**b**), fasting for 16h, and quantification of the area under the curve (AUC) (**c**), panels shown as mean ±SEM, control LFD n=9, *Ad-Tmem120a*$^{-/-}$ LFD n=8, control HFD n=9, *Ad-Tmem120a*$^{-/-}$ n=10. **d**, Insulin tolerance test (**ITT**) in control and *Ad-Tmem120a*$^{-/-}$ males on LFD vs HFD, fasting for 6h, shown as mean ±SEM, control LFD n=9, *Ad-Tmem120a*$^{-/-}$ LFD n=9, control HFD n=10, *Ad-Tmem120a*$^{-/-}$ n=9. *P* values: by unpaired two-sided Student's *t*-test. Source data are provided as a Source Data file.

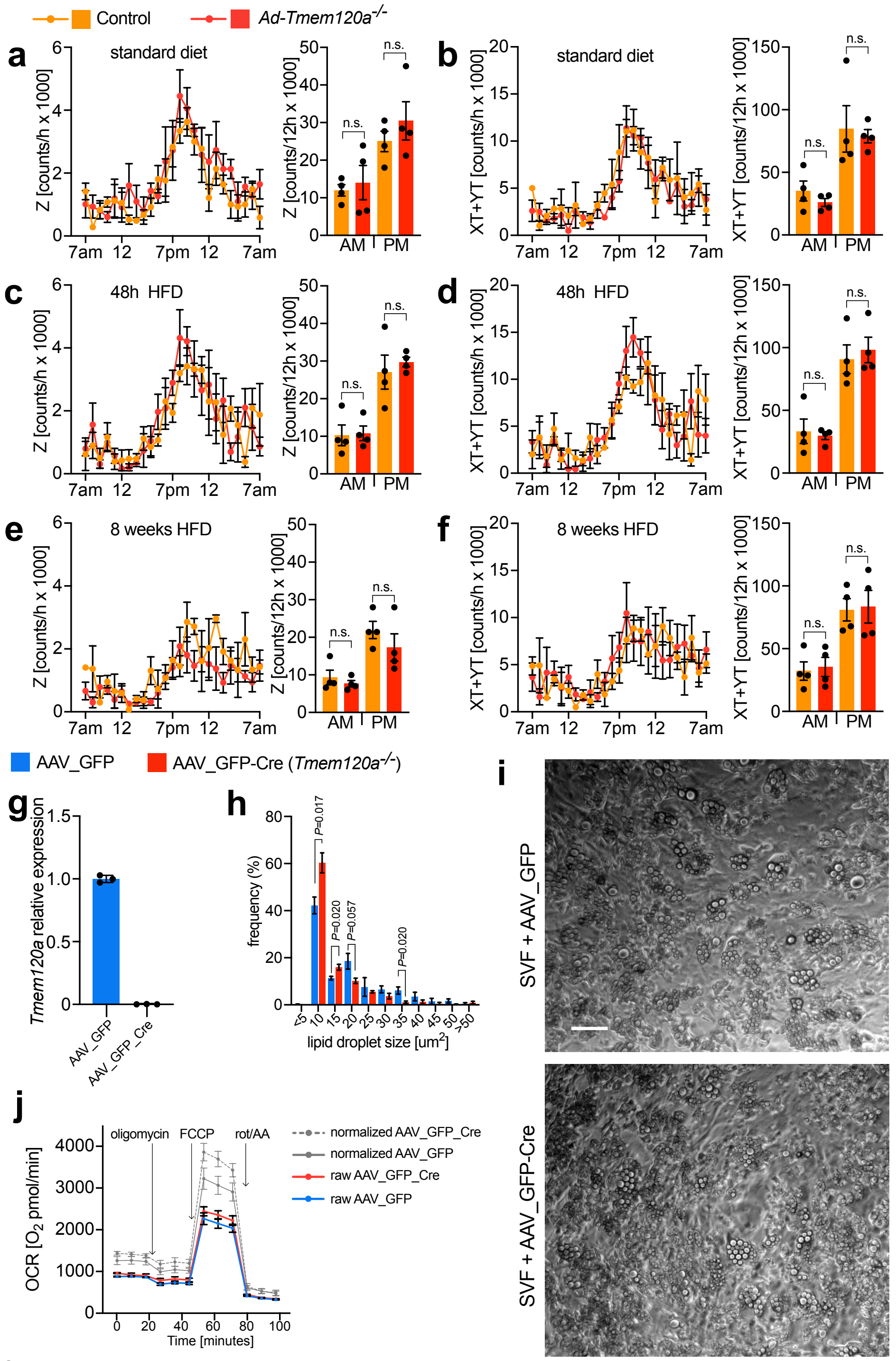

Supplementary Fig. 4

a and **b**, Mouse movement in indirect calorimetry chambers on standard diet. Activity data in z dimension (**a**) and activity in XY dimensions on (**b**) shown as number of infrared beams disruption during 12h cycles. Line graphs show a representative 24h trace and bar graph show the average from 48h. **c** and **d**, Mouse movement in indirect calorimetry chambers after 48h on high-fat diet. Activity data in z dimension (**c**) and activity in XY dimensions on (**d**) shown as number of infrared beams disruption during 12h cycles. Line graphs show representative mean ±SEM 24h trace and bar graph show the average from 48h. **e** and **f**, Mouse movement in indirect calorimetry chambers after 48h on high-fat diet. Activity data in z dimension (**e**) and activity in XY dimensions on (**f**). HFD control n=4, HFD *Ad-Tmem120a*$^{-/-}$ n=4. **g**, Expression by qPCR of *Tmem120a* in differentiated SVF cells by qPCR in cells infected with AAV-GFP (Control) and AAV_GFP-Cre (*Tmem120*$^{-/-}$), n=3 separate AAV infection prep per group. **h**, Quantification of lipid droplet size in differentiated SVF cells infected AAV-GFP (Control) – blue bars and AAV_GFP-Cre (*Tmem120*$^{-/-}$) – red bars, the sizes of the lipid droplets were assigned to size bins and are shown as frequency, area in μm^2, shown as mean ±SEM, n=4 separate AAV infections per group, *P* values calculated by unpaired, two-sided Student's t test. **i**, Representative images of lipid droplets in *in vitro* differentiated SVFs, scale bars 10 μm. in total 8 pictures were analysed per group across 4 separate AAV infections per group (i.e., GFP vs GFP-Cre). **j**, Comparison of total protein-normalized (grey lines) and raw (blue/red) values of oxygen consumption rates (OCR) in SVF cells differentiated into adipocytes, isolated from lox/lox animals infected with AAV_GFP_Cre and AAV_GFP. Mitochondrial stress experiment in the presence of oligomycin, FCCP, and rotenone with antimycin A (rot/AA), n=6 separate AAV infection per group as described in Fig 3. Source data are provided as a Source Data file.

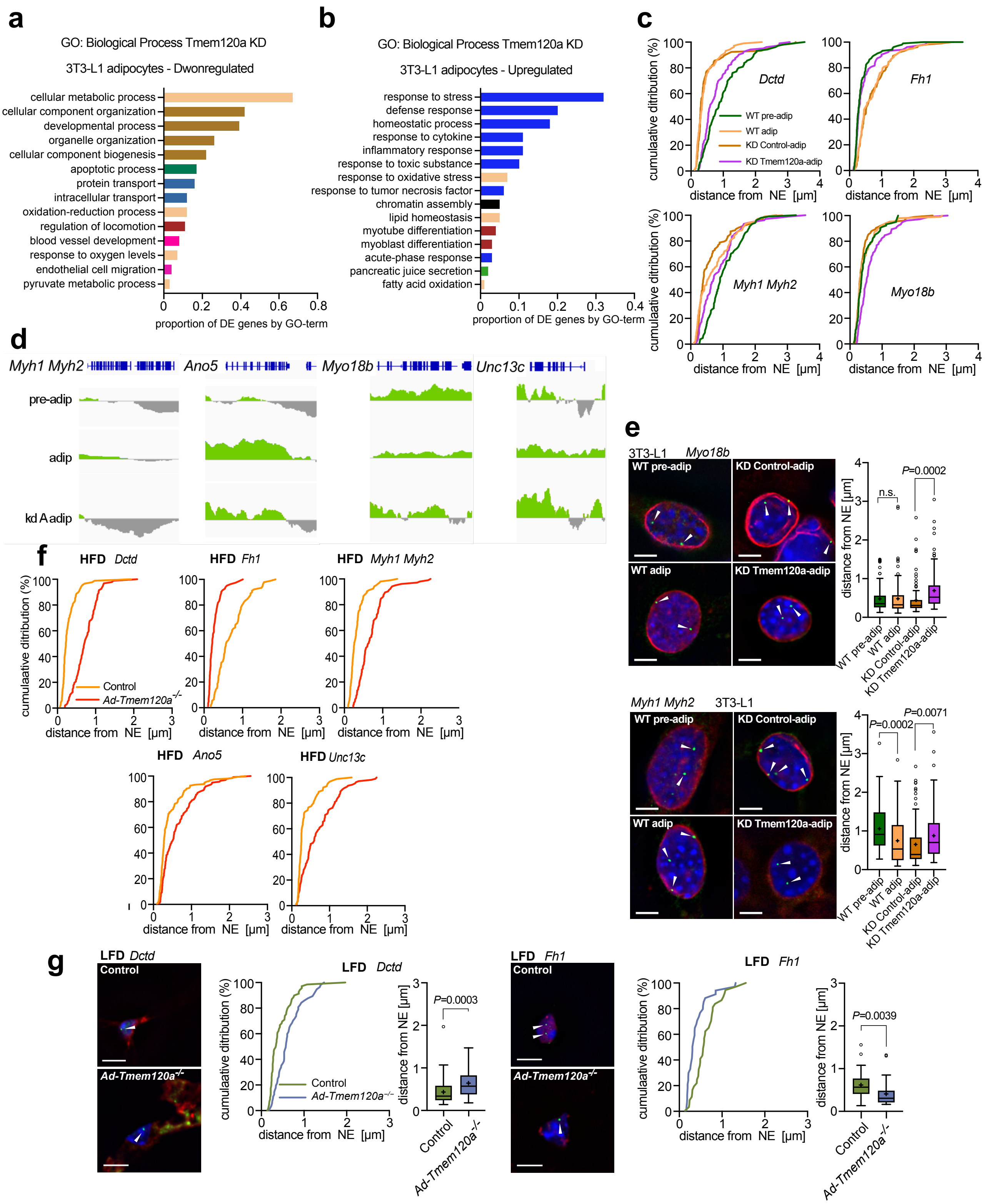

Supplementary Fig. 5

a and **b**, Gene ontology (GO): Biological Process of genes downregulated (**a**) and upregulated (**b**) by microarray in *Tmem120a* knockdown 3T3-L1 adipocytes. **c**, Cumulative distribution plots of gene distance from NE for data from FISH on 3T3-L1 adipogenesis system presented in main Fig. 5. **d**, 3T3-L1 DamID traces for additional genes tested. **e**, *Myh1/Myh2* and *Myo18b* FISH and box plot graph showing quantification of distance from NE in 3T3-L1 system, shown as Tukey box plot representing median, cross on the box represents mean, bounds of box represents interquartile of the data, whiskers representing minima/maxima excluding outliers and dots represents outliers of more than 2/3 times of upper quartile, n=104 loci per group. **f**, Quantification of genes distance from NE in iWAT from control and *Ad-Tmem120a*$^{-/-}$ mice, shown as cumulative distribution. **g**, FISH in low fat diet (LFD) mouse iWAT and quantification graphs of distance from NE for Dctd WT n=70 loci and KO n=58, for Fh1 WT n=60 and KO n=53 presented as cumulative distribution graphs and as Tukey box plot representing median, cross on the box represents mean. Scale bars 5 μm. *P* values calculated by unpaired, two-sided Student's *t*-test. Source data are provided as a Source Data file.

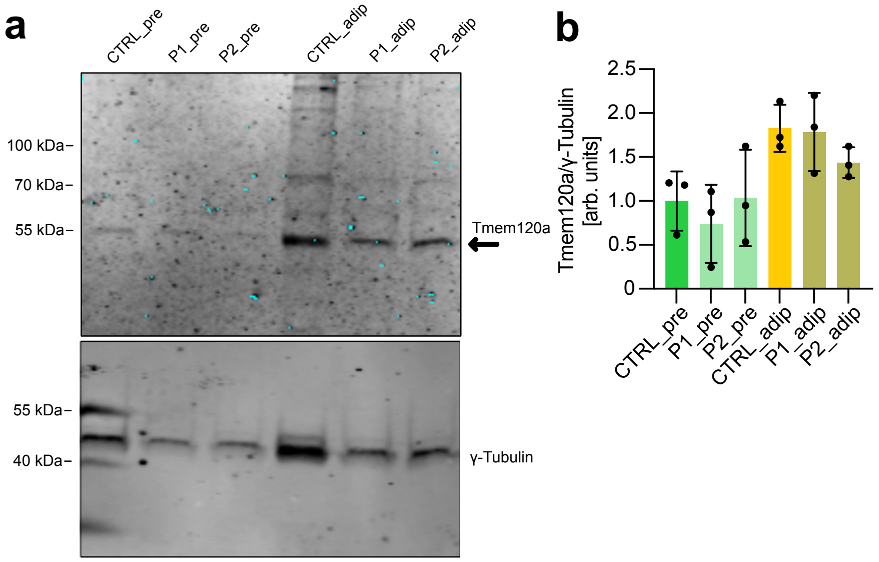

Supplementary Fig. 6

a, Western blotting detection Tmem120a protein expression in primary pre-adipocytes (_pre) from healthy control (CTRL) and FPLD2 patients (P1 and P2) as well as in differentiated adipocytes (_adip). **b**, Quantification of TMEM120A signal intensity normalised to γ-Tubulin signal, shown as mean from 3 experiments, error bars represent SD. Source data are provided as a Source Data file.
