# Supplementary material for: Genomic loci mispositioning in Tmem120a knockout mice yields latent lipodystrophy
Source: Nat Commun. 2022 Jan 13;13:321. doi: 10.1038/s41467-021-27869-2 (PMC8758788; doi:10.1038/s41467-021-27869-2)
Supplement: Supplementary file 3 — Description of additional Supplementary File [file 41467_2021_27869_MOESM3_ESM.pdf]

<u>Descriptions of additional supplementary data files</u>

Supplementary Data S1

Summary of the *Ad-Tmem120a-/-* phenotype in females on LFD vs. HFD.

Supplementary Data S2

RNA-Seq gene expression changes in knockout inguinal WAT.

Supplementary Data S3

Microarray and DamID data from 3T3-L1 knockdown and differentiation with both *Tmem120a* and *Tmem120a*/*b* double knockdowns.

Supplementary Data S4

Largest RNA-Seq and lncRNA expression changes in knockout inguinal WAT and behaviour in other datasets.

Supplementary Data S5

miRNA expression changes and corresponding gene targets.
