# Supplementary material for: Genomic loci mispositioning in Tmem120a knockout mice yields latent lipodystrophy
Source: Nat Commun. 2022 Jan 13;13:321. doi: 10.1038/s41467-021-27869-2 (PMC8758788; doi:10.1038/s41467-021-27869-2)
Supplement: Supplementary file 9 — Reporting Summary [file 41467_2021_27869_MOESM9_ESM.pdf]

nature research

Corresponding author(s): Eric C Schirmer

Last updated by author(s): Nov 29, 2021

Reporting Summary

Nature Research wishes to improve the reproducibility of the work that we publish. This form provides structure for consistency and transparency in reporting. For further information on Nature Research policies, see our Editorial Policies and the Editorial Policy Checklist.

Statistics

For all statistical analyses, confirm that the following items are present in the figure legend, table legend, main text, or Methods section.

n/a	Confirmed	
☐	☒	The exact sample size (*n*) for each experimental group/condition, given as a discrete number and unit of measurement
☐	☒	A statement on whether measurements were taken from distinct samples or whether the same sample was measured repeatedly
☐	☒	The statistical test(s) used AND whether they are one- or two-sided<br>*Only common tests should be described solely by name; describe more complex techniques in the Methods section.*
☒	☐	A description of all covariates tested
☐	☒	A description of any assumptions or corrections, such as tests of normality and adjustment for multiple comparisons
☐	☒	A full description of the statistical parameters including central tendency (e.g. means) or other basic estimates (e.g. regression coefficient) AND variation (e.g. standard deviation) or associated estimates of uncertainty (e.g. confidence intervals)
☐	☒	For null hypothesis testing, the test statistic (e.g. *F*, *t*, *r*) with confidence intervals, effect sizes, degrees of freedom and *P* value noted<br>*Give P values as exact values whenever suitable.*
☒	☐	For Bayesian analysis, information on the choice of priors and Markov chain Monte Carlo settings
☒	☐	For hierarchical and complex designs, identification of the appropriate level for tests and full reporting of outcomes
☒	☐	Estimates of effect sizes (e.g. Cohen's *d*, Pearson's *r*), indicating how they were calculated

Our web collection on statistics for biologists contains articles on many of the points above.

Software and code

Policy information about availability of computer code

Data collection	Seahorse, Wave Controler Software, v. 2.4 for the XF24 Extracellular Analyzer, Agilent Technologies.<br>Phnenomaster controller software v.6.1.9, TSE, Indirect Calorimetry.<br>Multiplate reader, Multimode Plate Reader - Molecular Devices M5, SoftMax Pro software, Version, 6.1.<br>ImageJ -Micromanager v1.4.22, image acquisition software.<br>MetaMorph, Molecular Devices, Version: 7.8 - image acquisition software.<br>qPCR - LightCycler 480 Software, Version 1.5, Roche
Data analysis	ImageJ, Fiji, NIH, Schneider et al., 2012, Version: 2.1.0 (RRID:SCR_00228),R, Project,Version: 1.46.0, (RRID:SCR_001905)<br>BioConductor, HTqPCR package, Version: 1.48, ref. Dvinge and Bertone, 2009, https://www.bioconductor.org/packages/release/bioc/html/HTqPCR.html, (RRID:SCR_003375)<br>BioConductor, DNAcopy package, ref. Seshan and Olshen, 2016, https://www.bioconductor.org/packages/release/bioc/html/DNAcopy.html, (RRID:SCR_012560)<br>BioConductor, GOstats, ref. Falcon and Gentleman, 2007, https://bioconductor.org/packages/release/bioc/html/GOstats.html, (RRID:SCR_008535)<br>Limma Smyth, 2005, https://bioconductor.org/packages/release/bioc/html/limma.html, (RRID:SCR_010943)<br>DESeq2, Version: 1.24.0<br>R, Tximport package, Versio: 1.22<br>STAR, Version: 2.5.3.a, (RRID:SCR_015899)<br>BEDtools, Version: 2.2.7, (RRID:SCR_006646)<br>SAMtools, Version: 1.9, (RRID:SCR_002105)<br>Salmon, Version: 0.13.1 (RRID:SCR_017036)

g:Profiler, ref. Raudvere et al., 2019, https://biit.cs.ut.ee/gprofiler/gost, (RRID:SCR_006809)
TissueEnrich, ref. Jain and Tuteja, 2019, https://tissueenrich.gdcb.iastate.edu
Affinity Designer, Serif, Version: 1.8, (RRID:SCR_016952)
Photoshop Adobe, Version: 6, (RRID:SCR_014199)
Imaris – Bitplane Oxford Instruments, Version: 8, (RRID:SCR_007370)
AutoQuant X3, Media Cybernetics, Version: 3.1.3, (RRID:SCR_002465)

For manuscripts utilizing custom algorithms or software that are central to the research but not yet described in published literature, software must be made available to editors and reviewers. We strongly encourage code deposition in a community repository (e.g. GitHub). See the Nature Research guidelines for submitting code & software for further information.

Data

Policy information about availability of data

All manuscripts must include a data availability statement. This statement should provide the following information, where applicable:

- Accession codes, unique identifiers, or web links for publicly available datasets
- A list of figures that have associated raw data
- A description of any restrictions on data availability

Raw datasets and bioinformatic analysis of data used in this study are available trough public repositories. RNA-Seq datasets have been made available through the Gene Expression Omnibus (GEO) repository at NCBI under accession number GSE147016 https://www.ncbi.nlm.nih.gov/geo/query/acc.cgi?acc=GSE147016. DamID raw data is available through the Gene Expression Omnibus (GEO) repository at NCBI with accession number GSE150507 https://www.ncbi.nlm.nih.gov/geo/query/acc.cgi?acc=GSE150507. Microarray raw data are available under accession number 150022 https://www.ncbi.nlm.nih.gov/geo/query/acc.cgi?acc=GSE150022.

Field-specific reporting

Please select the one below that is the best fit for your research. If you are not sure, read the appropriate sections before making your selection.

[x] Life sciences [ ] Behavioural & social sciences [ ] Ecological, evolutionary & environmental sciences

For a reference copy of the document with all sections, see nature.com/documents/nr-reporting-summary-flat.pdf

Life sciences study design

All studies must disclose on these points even when the disclosure is negative.

Sample size

From our independent biased prediction of sample size, assuming that we will see differences at significance level of 5%, statistical power of 80%, allowing detection in differences in fat mass and metabolic parameters between groups with a sensitivity limit of +/- 15%, we have calculated the numbers of mice needed maximally as 10 per genotype per condition. However, due to the complex nature of Cre/Lox mouse mating and low usable genotype offspring number, we defined our sample size as minimum of 3 and maximum of 10. For RNA-seq and high throughput qPCR 5 animals per group were used. The sample size i.e. the number of nuclei analyzed for FISH experiments on culture cells and tissues was estimated a priori by statistical power analysis for t test by software suit G*power v3.1. Assuming alpha error probability at 5% and power of 80% and effect size at 0.5, we calculated n number of 50 nuclei were appropriate to analyze per condition/genotype/animal.

Data exclusions

No data were excluded in this research.

Replication

Two separate animal experiments were conducted for animal study; with both: males and females. All data from these experiments were used in the analysis. Animal tissues were analyzed in triplicates in relevant experiments. For RNA-seq and high-throughput qPCR - miRNA we used 5 animals per group with no technical replicates. FISH experiments on cells were performed in two technical replicates per gene loci and the replication attempt was successful.

Randomization

Animals were assigned by random to dietary groups in high- , low - fat diet experiment. Cell lines and primary cell did not require randomization as they were all analyzed in pairs of pre-adipocyets vs adipocytes.

Blinding

Experimenter was blinded to genotype and diet during glucose tolerance and insulin tolerance experiments as well as during TD-NMR experiments. Experimenters were also blinded to genotype and diet during histological tissue analysis and while performing analysis of FISH experiment on mouse adipose tissue. Similarly, experimenter was blinded to genetic status of cultured cells used in FISH experiments. Blinding was not possible for analysis of adpiocytes vs pre-adipocytes as lipid droplets are visible on microscope pictures.

Reporting for specific materials, systems and methods

We require information from authors about some types of materials, experimental systems and methods used in many studies. Here, indicate whether each material, system or method listed is relevant to your study. If you are not sure if a list item applies to your research, read the appropriate section before selecting a response.

Materials & experimental systems

n/a	Involved in the study	
	x	Antibodies
	x	Eukaryotic cell lines
x		Palaeontology and archaeology
	x	Animals and other organisms
	x	Human research participants
x		Clinical data
x		Dual use research of concern

Methods

n/a	Involved in the study	
x		ChIP-seq
x		Flow cytometry
x		MRI-based neuroimaging

Antibodies

Antibodies used	rabbit Lamin A antibodies , identifier: 3262, (homemade, available upon request)<br>rabbit Tmem120a, identifier: R1196, (homemade, available upon request)<br>rabbit Tmem120b, identifier: R3246, (homemade, available upon request)<br>rabbit anti-GFP (Invitrogen, A11122)<br>mouse gamma Tubulin (SIGMA, T6657)<br>donkey anti-rabbit IRDye 800CW - (LiCor, Cat: 926-32213, Lot: C61012-02)<br>donkey anti-mouse IRDye 680RD - LiCor, Cat: 926-68073, Lot: C90821-03<br>Donkey anti-rabbit 594 Alexa Fluor - Thermo Fisher, A21207<br>488 Alexa Fluor conjugated anti-digoxigenin - Jackson ImmunoResearch, 200-542-156
Validation	Lamin A antibodies were validated in Schirmer et. al., 2001, https://doi.org/10.1083/jcb.153.3.479.<br>Tmem120a were validated in Batrakou et al., 2015, https://doi.org/10.1371/journal.pone.0127712.<br>Tmem120b were validated in this paper.<br>anti-GFP were validated by Invitrogen, "Antibody specificity was demonstrated by detection of different targets fused to GFP tag in transiently transfected lysates tested." https://www.thermofisher.com/antibody/product/GFP-Antibody-Polyclonal/A-11122.<br>mouse gamma Tubulin were validated by Keuling et al., 2009, https://doi.org/10.1371/journal.pone.0006651

Eukaryotic cell lines

Policy information about cell lines

Cell line source(s)	3T3-L1, ATCC, CL-173 (RRID_0123)<br>HEK293FT, ATCC, PTA-5077, (RRID_6911)
Authentication	Cells were not authenticated
Mycoplasma contamination	Cell lines used in this study tested negative for mycoplasma contamination. All cell batches were regularly tested for mycoplasma by standard PCR.
Commonly misidentified lines<br>(See ICLAC register)	No commonly misidentified cell lines were used in this study.

Animals and other organisms

Policy information about studies involving animals; ARRIVE guidelines recommended for reporting animal research

Laboratory animals	Mouse C57BL/6NTac, Tmem120A loxP/loxP, Taconic Artemis, C57BL/6NTac-Tmem120atm3635.1Arte. Males and females were used at the age of 3-6 months.<br>Mouse C57BL/6JCrl, Adipoq_Cre, Eguchi et al., 2011, Tg(Adipoq-Cre)1Evdr, (RRID_IMSR_JAX:028020). Males and females were used at the age of 3-6 months.
Wild animals	The study did not involve wild animals.
Field-collected samples	The study did not involved the field-collected samples.
Ethics oversight	Experiments involving animals were approved by the University of Edinburgh ethical committee and Home Office, UK under project licenses granted to ECS and NMM.

Note that full information on the approval of the study protocol must also be provided in the manuscript.

Human research participants

Policy information about studies involving human research participants

Population characteristics	Patients and healthy donor sample were female, mean age of 30. FPLD2 patients carried R482Q mutation in LMNA

	gene.
Recruitment	Human preadipocytes from familial partial lipodystrophy type 2 patients were from the BioLaM biobank located at the CNR Institute of Molecular Genetics Unit of Bologna. Healthy control comes from the neck adipose tissue of patient undergoing aesthetic surgery had been collected according to the same local and EU ethical rules.
Ethics oversight	Rizzoli Orthopedic Institute Ethical Committee approval, No. 0018250–2016, Bologna, Italy

Note that full information on the approval of the study protocol must also be provided in the manuscript.
